# Taste Ratings of Healthier Main and Side Dishes among 4-to-8-Year-Old Children in a Quick-Service Restaurant Chain

**DOI:** 10.3390/nu13020673

**Published:** 2021-02-19

**Authors:** Sara Tauriello, Lily McGovern, Brianna Bartholomew, Leonard H. Epstein, Lucia A. Leone, Juliana Goldsmith, Elizabeth Kubiniec, Stephanie Anzman-Frasca

**Affiliations:** 1 Department of Pediatrics, Jacobs School of Medicine and Biomedical Sciences, University at Buffalo, Buffalo, NY 14214, USA; sarataur@buffalo.edu (S.T.); lilymcgo@buffalo.edu (L.M.); lhenet@buffalo.edu (L.H.E.); jmgoldsm@buffalo.edu (J.G.); emkubini@buffalo.edu (E.K.); 2 Independent Health Foundation, Buffalo, NY 14221, USA; Brianna.Bartholomew@independenthealth.com; 3 Center for Ingestive Behavior Research, University at Buffalo, Buffalo, NY 14214, USA; lucialeo@buffalo.edu; 4 Department of Community Health and Health Behavior, School of Public Health and Health Professions, University at Buffalo, Buffalo, NY 14214, USA

**Keywords:** restaurants, children, food preferences, diet quality

## Abstract

Restaurants are regular eating environments for many families. Children’s consumption of restaurant foods has been linked with poorer diet quality, prompting emerging research examining strategies to encourage healthier eating among children in restaurants. Although taste is a primary determinant of restaurant meal choices, there is a lack of research considering children’s perspectives on the taste of different healthier kids’ meal options. The current study sought to examine, via objective taste testing, children’s liking of and preference for healthier kids’ meal options at a quick-service restaurant (QSR) and to describe bundled kids’ meals with evidence of both taste acceptability and consistency with nutrition guidelines. Thirty-seven 4-to-8-year-old children completed taste tests of ten healthier main and side dish options. Liking and preference were assessed using standard methods after children tasted each food. Children also reported their ideal kids’ meal. Results show the majority of children liked and preferred three main (turkey sandwich, chicken strips, peanut butter/banana sandwich) and side dishes (yogurt, applesauce, broccoli), with rank order differing slightly by age group. Accepted foods were combined into 11 bundles meeting nutritional criteria. Results highlight healthier kids’ meals with evidence of appeal among children in a QSR. Findings can inform future research and may increase the success of healthy eating interventions in these settings.

## 1. Introduction

Diets of children in the United States are generally energy dense, nutrient poor [1,2] and fail to meet national fruit and vegetable intake recommendations [3,4]. Early eating habits can have lasting implications, as food preferences tend to remain stable and persist into adulthood [5,6]. However, taste preferences are malleable and children can learn to like healthier foods that are available and become familiar [7,8,9]. Yet, this process relies upon regular exposure to healthier foods, which is not normative within many children’s eating environments, including restaurants. 

Restaurants have become a regular eating context for many families. On any given day, one-third of children visit quick-service restaurants (QSR) where they consume approximately one-third of their daily energy intake [10,11]. Restaurant meals tend to be higher in calories and lower in nutritional quality than meals prepared at home and few meet nutrition recommendations [12,13,14]. Consequently, consumption of restaurant meals has been linked with increased daily intake of energy, saturated fat, sugar and sodium among children [15,16]. Thus, restaurants are one setting where shifts in current eating behavior have the potential to help children create healthy eating habits. 

Industry trends suggest that restaurants are beginning to add healthier items to children’s menus [17]. Supporting this, a recent analysis of 66 large restaurant chains within the United States found newly added menu items had fewer calories than original items [18]. In 2011, The National Restaurant Association introduced the Kids LiveWell program, which aims to help parents and children select healthier options when dining out. Restaurants participating in the program must offer at least two meal and side dishes that meet established nutrition criteria and provide healthier beverages (milk, water and/or 100% fruit/vegetable juice) by default [19]. While participation in this program is voluntary, some localities have passed legislation requiring that children’s menus adhere to similar standards [20,21], although at present, such policies are the exception and not the rule. 

Restaurant-based research can help inform the continued development and implementation of approaches aiming to making healthy choices easier for families in restaurants. Strategies that have demonstrated some efficacy in restaurant-based intervention research include making healthier side dishes the default option [22,23,24], modifying the prominence of healthier options [25,26,27], offering incentives [27,28] and serving healthier foods prior to the main meal [29]. Although these strategies are supported by rigorous evidence from other domains [30,31,32], observed effects of these evidence-based strategies have generally been smaller in restaurant settings, which may be due to the high number of palatable, less nutritious competing alternatives and/or a lack of attention to the role of taste. If healthier options are infrequently purchased, restaurants may lack incentive to continue supplying them [33], highlighting a need for research with the potential to increase the effectiveness of these efforts. 

Given that taste is a primary determinant of restaurant food choices [25,33,34], it is important to understand the types of healthier options that children are likely to accept in restaurants. Efforts to increase the uptake of healthier children’s meals will likely be more successful if the promoted healthy meals have demonstrated evidence of acceptability among children. Yet to date, there is a dearth of research on children’s liking of and preferences for healthy kids’ meal options. Results from two nationally representative surveys of children found that the majority of 8-to-12-year-olds who reported ordering kids’ meals indicated that they would be likely to order a meal that came with vegetables or fruits [34,35] and that they would be open to receiving a fruit or vegetable side dish instead of French fries, or milk or water instead of soda with their meal [34]. However, these perspectives were collected outside of the restaurant setting and no research to date has objectively assessed taste acceptance in restaurant settings. In order to accelerate progress in promoting healthier eating among children in restaurants, research is needed to understand children’s responses to the taste of healthier kids’ meal options in these settings, including younger children who frequently eat at restaurants. 

The primary aim of the current study was to examine, via objective taste testing, liking of and preferences for healthier main and side dishes at two locations of a regional QSR chain among 4-to-8-year-old children who eat in restaurant settings regularly. In addition, as QSR menus frequently feature bundled kids’ meals, children’s perspectives on kids’ meal bundles were assessed and bundled meals that demonstrated both palatability (via child liking and preference) and consistency with nutrition recommendations are described. Findings have the potential to inform restaurant-based health promotion efforts by describing healthier kids’ meals likely to be accepted by families.

## 2. Methods

### 2.1. Participants

Participating children were recruited over a three-week period in Fall 2019 from two locations of Anderson’s Frozen Custard, a regional quick-service American restaurant in Buffalo, NY, known for its roast beef sandwiches and frozen custard. Families with children were approached at their table after ordering and received a verbal description of the study. To be eligible, families needed to meet the following criteria: English-speaking parent/legal guardian 18 years of age or older with an English-speaking child 4–8 years old who eats food from restaurants at least once per week and does not have allergies that preclude safe participation. This age range was selected to capture the target audience of kids’ menus. In instances where families had multiple children, each eligible child was able to participate if desired. Forty-three families were screened for participation. Thirty-eight children were eligible and 37 from 30 families (i.e., 7 sibling pairs) completed study procedures. Children’s ages ranged from 4–8 years; 92% were white and 8% multiracial (see Table 1 for demographics). The analytic sample is 37; however, one child was a vegetarian, so 36 children tasted and rated the two main dishes that contained meat. Children gave verbal (<7 years) or written (≥7 years) assent prior to participating in study procedures. This study was conducted according to the guidelines of the Declaration of Helsinki. All procedures were approved by the Institutional Review Board of the University at Buffalo (protocol ID 00003356; approved 4 April 2019).

### 2.2. Procedures

Trained study staff were present in each restaurant during dinner (4:30–8 p.m.) for 1–2 days per week during the three-week data collection period. Eligible families received information about study procedures and parents completed consent forms and a brief demographic survey following their meal. Participating children individually tasted and rated their liking and preference for five healthier main (avocado toast, baked potato with cheese and broccoli, grilled chicken strips with honey mustard and/or ranch dip, peanut butter and banana sandwich, turkey sandwich) and side dishes (applesauce, mango/peach/pineapple fruit cup, steamed broccoli, steamed spinach, strawberry yogurt) at a separate table. Children were also asked which main and side dish they would choose in a bundled kids’ meal and what beverage(s) they would like with that meal. Families received USD 10 for participation and children received a sticker.

Tested food options were selected based on operational feasibility, nutritional quality and likely appeal to children. In finalizing the list of potential options to be tested, the restaurant’s owner weighed in on feasibility and a registered dietitian (BB) checked each food option for compliance with the National Restaurant Association’s Kids LiveWell nutrition guidelines, using the guidelines for a full meal to check main and side dish combinations alongside beverage options of milk, 100% apple juice and water. Initial indicators of food appeal were also gathered from prior research [25,35] and conversations with children, and we strived to offer a variety among the final options tested (e.g., fruit, vegetable and dairy-based sides). 

### 2.3. Measures

Demographics. Parents indicated their child’s age, race/ethnicity and frequency of eating at restaurants, as well as their own marital status, education and annual household income. 

Main/Side Dish Liking. Liking was assessed using procedures adapted from Birch and colleagues [36]. Children were presented with a small portion of each study food one at a time and were asked to take a small bite. The order of study foods was randomized across children. Using a 3-point (4–5-year-olds) or a 5-point (6–8-year-olds) visual face scale, children were asked to indicate whether the food tasted yummy, just OK, or yucky (3-point scale), or really good, sort of good, just OK, sort of bad, or really bad (5-point scale) by placing their sample cup in front of the corresponding face after the tasting.

Main/Side Dish Preference. Rank-ordered preference was assessed using procedures adapted from Birch and colleagues [36]. Following completion of the liking assessment, cups containing a small portion of each sample food rated as yummy/really good were placed in front of the child. The child was then asked to indicate which of the yummy/really good foods they thought was the yummiest/best. The cup containing the indicated food was then removed from sight and the child was again asked to indicate which of the remaining foods was the yummiest/best until all of the yummy/really good foods had been ranked. This procedure was repeated for each liking category until every food received a rank order. Rankings ranged from 1–10, with one indicating the child’s most preferred food and ten indicating the least preferred.

Perspectives on Kids’ Meal Bundles. Next, 10 cups containing a small portion of each sample food were placed in front of the child in two separate groups: a group with the five main dishes and a group with the five side dishes. Children were then asked to indicate which of the main dish options and which of the side dish options they would most like to have in a bundled kids’ meal. After selecting the two foods, children were asked to indicate which beverage(s) they would like to have with their chosen meal (selecting all that applied from options of: apple juice, milk, water, other). 

### 2.4. Statistical Analyses

Data were analyzed using SAS Version 9.4 (Cary, NC, USA). To examine children’s preferences for tested main and side dishes, means of rank-ordered preferences for each food item were calculated overall and by age group (i.e., younger, 4–5 years old; older, 6–8 years old), with an a priori plan to describe the “top 3” dishes based on these means. Frequencies were calculated to examine children’s liking for each main and side dish option, overall and by age group. Liked dishes were defined as those with a rating of “yummy” for younger children and “sort of good” or “really good” for older children. Frequencies were also calculated for the main and side dish pairings children selected when asked about their ideal kids’ meal, as well as the beverage(s) that children indicated that they would like with that bundled meal. 

After examining these individual items, we explored bundled kids’ meals with evidence of both palatability and correspondence with nutritional criteria. First, we examined whether the top 3 healthier main and side dishes were also liked by the majority of children, in order to identify which tested healthier options were both preferred (over other options) and well-liked. Main and side dishes meeting both criteria (i.e., preferred and liked by the majority) were bundled with one another and with each of the three healthier beverages recommended by the Kids’ LiveWell program: 100% juice (e.g., apple), milk and water. Iterations of these well-accepted bundles were retained as examples of palatable and nutritious kids’ meals if they also met Kids LiveWell nutritional criteria (i.e., ≤550 total calories, 10% saturated fat, 700 mg sodium, 15 g added sugars, 0 g trans-fat). 

## 3. Results

### 3.1. Main and Side Dish Preference

Overall, children’s most preferred main dishes (i.e. those lowest in average rank order) were the grilled chicken strips (M = 4.8, SD = 2.5), turkey sandwich (M = 4.9, SD = 2.1) and peanut butter and banana sandwich (M = 5.6, SD = 2.6). Among younger children, the peanut butter and banana sandwich (M = 4.9, SD = 2.1) was the most preferred, followed by the turkey sandwich (M = 5.2, SD = 2.3) and chicken strips (M = 5.6, SD = 3.2). For older children, the most preferred main dishes were the chicken strips (M = 4.4, SD = 2.0), turkey sandwich (M = 4.8, SD = 2.1) and peanut butter and banana sandwich (M = 5.8, SD = 2.8), respectively. Among examined side dishes, the strawberry yogurt (M = 3.2, SD = 2.8), applesauce (M = 4.5, SD = 2.8) and steamed broccoli (M = 4.8, SD = 2.5) were the most preferred overall, and for both younger and older children specifically. Full preference data are shown in Table 2.

### 3.2. Main and Side Dish Liking

Across both age groups, more than half of the children liked the turkey sandwich (66.7%), grilled chicken strips (63.9%) and peanut butter and banana sandwich (56.8%). For the examined side dishes, the majority of children liked the strawberry yogurt (83.8%), applesauce (75.7%), steamed broccoli (67.6%) and mixed fruit cup (62.2%). Results were similar when looking specifically at younger and older children; full liking data are shown in Table 2. 

### 3.3. Kids’ Meal Bundle Perspectives

When selecting specific foods that they would like to have in a bundled kids’ meal, children’s most commonly named main dish was the grilled chicken strips (35.1%), which they chose to pair with the strawberry yogurt (61.5%), steamed broccoli (15.4%), mixed fruit cup (15.4%), or applesauce (7.7%). The most commonly named side was strawberry yogurt (56.8%), which was most often paired with chicken strips (38.1%), followed by peanut butter and banana sandwich (28.6%), avocado toast (23.8%), baked potato (4.8%) and turkey sandwich (4.8%). When asked which drinks that they would like with their kids’ meal, children selected water (73.0%), apple juice (62.2%), other (62.2%; e.g., chocolate milk, soft drinks) and milk (46.0%). 

### 3.4. Kids’ Meal Bundles Meeting Kids LiveWell Guidelines

Kids’ meal bundles demonstrating evidence of both palatability and nutritional quality are shown in Table 3.

## 4. Discussion

Findings from these taste tests in QSR settings suggest that the top main dishes were the turkey sandwich, grilled chicken strips and peanut butter and banana sandwich, with some variation in the order depending on measure (i.e., liking, preference) and age group. On average, younger children preferred the peanut butter and banana sandwich the most, whereas older children preferred the chicken strips followed by the turkey sandwich. The most preferred side dishes among both age groups included the strawberry yogurt, applesauce and steamed broccoli. Each of these six preferred food options was liked by the majority of children, both overall and by age group. In addition, the fruit cup was also well-liked, particularly among older children.

These findings indicate that children in both age groups accepted healthier main and side dish options in these QSR settings. Observed differences in the ranking of top dishes between age groups may reflect differential exposure to foods as children age, as prior work has shown that children like and prefer foods that are familiar [7,8,9]. However, across both age groups, the majority of children liked all three of the top main and side dish options. Their nomination in children’s selected kids’ meal bundles suggests that grilled chicken strips and yogurt may be particularly popular. Inclusion of well-liked options in healthier kids’ meal bundles may increase the likelihood that they are selected when competing with palatable, familiar alternatives, increasing the likelihood of impacts on children’s diets, as well as the continued offering of healthier options on kids’ menus.

Using the main and side dishes that met criteria for further consideration based on their appeal during taste testing, 11 meal bundles were created that meet Kids LiveWell nutrition guidelines [19]. Although these menu items’ nutritionals are specific to the participating QSR, the bundles suggest a variety of acceptable fruit, vegetable and protein combinations are possible, enabling bundles to be tailored to particular consumer demands, dietary restrictions (e.g., vegetarian) and operational needs (e.g., utilizing foods already available in the pantry; [33]). These bundles highlight some possible meals that could be incorporated in health promotion efforts aimed at promoting heathier kids’ meals in ways that will be appealing to families and feasible for restaurants.

Limitations of the current study include the small, homogenous, convenience sample, which limits generalizability of findings, as well as the greater number of older versus younger children. Although descriptive analyses were conducted by age group to detect patterns among younger and older children specifically, the small sample size precludes further group analysis. Future work with a larger sample could address these concerns and further inform acceptance of healthier kids’ meal options by different demographics (e.g., socioeconomic status). Furthermore, only healthier main and side dish options were tested. While this was necessary to avoid presenting more choices than would be developmentally appropriate for the participating children, future work can build on these findings by examining the acceptance of healthier options when directly compared to popular alternatives (e.g., chicken tenders, French fries). Children’s perspectives on meal bundles were also assessed using a single item for each meal component (i.e., main dish, side dish, beverage), and more work can be done to further understand how children perceive different combinations of foods or food types in these settings.

In addition to underscoring the importance of children’s perspectives, prior restaurant-based work has also demonstrated that parents may play a role in some children’s meal choices [25,37]. Examination of parents’ perceptions of simulated children’s menus outside of the restaurant setting has suggested that parents may prefer menus with low cost, multiple side dishes, a healthier entrée and a small upcharge for sugar-sweetened beverages [38]. However, more work is needed within restaurants to further elucidate acceptability of healthier kids’ meal options to parents, as well as whether the promotion of accepted healthier main and side dishes impacts children’s meal orders and dietary intake. 

Children’s consumption in restaurant settings continues to diverge from nutritional recommendations. As taste has been indicated as the primary determinant of restaurant meal choices, it is important to understand the types of healthier foods that children will accept in these settings. The current work utilized objective taste-testing to shed light on potential healthier options that children may find palatable in a QSR. Such findings can inform future research and increase the potential success of healthy eating interventions in these settings.

## Figures and Tables

**Table 1:** Demographic characteristics of participating families ^a^.

	Overall	Younger Children	Older Children
	(*n* = 37)	(*n* = 11)	(*n* = 26)
Child			
Race			
White	34 (91.9)	10 (90.9)	24 (92.3)
Multiracial	3 (8.1)	1 (9.1)	2 (7.7)
Frequency child eats food at a restaurant	
A few times per month ^b^	4 (10.8)	0 (0.0)	4 (15.4)
1–3 times per week	32 (86.5)	10 (90.9)	22 (84.6)
4+ times per week	1 (2.7)	1 (9.1)	0 (0.0)
Eligible for free- or reduced-price	
school meals	6 (16.22)	2 (18.18)	4 (15.4)
Parent			
Relationship to child		
Mother	25 (67.6)	8 (72.7)	17 (65.4)
Father	12 (32.4)	3 (27.3)	9 (34.6)
Marital Status		
Married	30 (81.1)	9 (81.8)	21 (80.8)
Divorced	3 (8.1)	0 (0.0)	3 (11.5)
Never married	3 (8.1)	2 (18.2)	1 (3.9)
Living with a partner	1 (2.7)	0 (0.0)	1 (3.9)
Parent Education		
High school graduate	1 (2.7)	0 (0.0)	1 (3.9)
Some college	4 (10.8)	0 (0.0)	4 (15.4)
Associates degree	2 (5.4)	1 (9.1)	1 (3.9)
Bachelor’s degree	9 (24.3)	3 (27.3)	6 (23.1)
Graduate degree	21 (56.8)	7 (63.6)	14 (53.9)
Annual Household Income	
>USD 50,000	3 (8.1)	1 (9.1)	2 (7.7)
USD 50,000–USD 99,999	9 (24.3)	2 (18.2)	7 (26.9)
USD 100,000+	22 (59.5)	8 (72.7)	14 (53.9)
Prefer not to answer	3 (8.1)	0 (0.0)	3 (11.5)

Note. ^a^ Values expressed as *n* (%). Child gender was not assessed in the demographic survey; however, both boys and girls participated. ^b^ All parents reported that their child eats at restaurants at least once per week during screening, but four reported a slightly lower frequency when asked about the child’s restaurant dining in the study survey.

**Table 2:** Children’s average ranked preference and self-reported liking of each tasted main and side dish.

	Overall (*n* = 37)	Younger Children (*n* = 11)	Older Children (*n* = 26)
Rank-ordered preference, M(SD) ^a^ Main dishes			
Grilled Chicken Strips	4.8 (2.5) ^c^	5.6 (3.2)	4.4 (2.0) ^c^
Turkey Sandwich	4.9 (2.1) ^c^	5.2 (2.3)	4.8 (2.1) ^c^
Peanut Butter/Banana Sandwich	5.6 (2.6)	4.9 (2.1)	5.8 (2.8)
Baked Potato	6.7 (2.8)	7.5 (2.7)	6.4 (2.8)
Avocado Toast	7.3 (2.7)	7.6 (1.9)	7.2 (3.0)
Side dishes			
Strawberry Yogurt	3.2 (2.8)	3.0 (2.5)	3.3 (3.0)
Applesauce	4.5 (2.8)	4.2 (3.3)	4.7 (2.7)
Steamed Broccoli	4.8 (2.5)	4.5 (2.4)	5.0 (2.6)
Mixed Fruit Cup	5.1 (2.6)	5.4 (3.1)	5.0 (2.4)
Steamed Spinach	7.8 (2.1)	7.1 (2.5)	8.1 (1.8)
Liking, *n*(%) who liked the dish ^b^ Main dishes			
Turkey Sandwich	24 (66.7) ^c^	6 (54.6)	18 (72.0) ^c^
Grilled Chicken Strips	23 (63.9) ^c^	6 (54.6)	17 (68.0) ^c^
Peanut Butter/Banana Sandwich	21 (56.8)	6 (54.6)	15 (57.7)
Baked Potato	15 (40.5)	4 (36.4)	11 (44.0)
Avocado Toast	12 (32.4)	3 (27.3)	9 (34.6)
Side dishes			
Strawberry Yogurt	31 (83.8)	9 (81.8)	22 (84.6)
Applesauce	28 (75.5)	8 (72.7)	20 (76.9)
Steamed Broccoli	25 (67.6)	9 (81.8)	16 (61.5)
Mixed Fruit Cup	23 (62.2)	5 (45.5)	18 (69.2)
Steamed Spinach	10 (27.0)	4 (36.4)	6 (23.1)

Note. ^a^ Rankings ranged from 1–10, with lower numbers indicating greater preference. ^b^ Liking was reported on 3- or 5-point scales, depending on child age and is operationalized here as the percentage of children who liked each dish, defined as providing a rating of yummy (younger children) or very good/sort of good (older children). ^c^ Sample size is *n* − 1 as one participating child was vegetarian.

**Table 3:** Kids’ meal combinations meeting Kids LiveWell guidelines (i.e., ≤550 cal, ≤10% saturated fat, ≤700 mg sodium, ≤15g added sugars, 0 g trans-fat) ^a^.

	Total Calories	Total Fat (g(%))	Saturated Fat (g(%))	Sodium (mg)	Sugar (g(%))	Added Sugars (g)
Chicken strips with ranch dressing, with:						
Yogurt + apple juice	420	15.5 (33.0)	2.0 (4.0)	515	33.0 (31.0)	6.0
Steamed broccoli + apple juice	360	13.5 (34.0)	1.0 (3.0)	460	25.0 (28.0)	1.0
Turkey sandwich, with:						
Yogurt + water	260	4.5 (16.0)	0.5 (1.7)	495	13.0 (20.0)	9.0
Yogurt + 1% milk	360	6.5 (16.0)	2.5 (6.3)	595	24.0 (27.0)	9.0
Applesauce + water	220	2.5 (10.0)	0.0 (0.0)	415	15.0 (27.0)	4.0
Applesauce + 1% milk	320	4.5 (13.0)	1.5 (4.2)	515	26.0 (33.0)	4.0
Steamed broccoli + water	200	2.5 (11.0)	0.0 (0.0)	440	5.0 (10.0)	4.0
Steamed broccoli + 1% milk	300	4.5 (14.0)	1.5 (4.5)	540	16.0 (21.0)	4.0
Peanut butter and banana sandwich, with:						
Yogurt + water	550	15.0 (25.0)	3.0 (5.0)	545	28.0 (20.0)	8.0
Applesauce + water	510	13.0 (23.0)	2.0 (4.0)	465	30.0 (24.0)	3.0
Steamed broccoli + water	490	13.0 (24.0)	2.0 (4.0)	490	20.0 (16.0)	3.0

Note. ^a^ Provided nutrition information is specific to participating QSR restaurant chain. Values reflect the total in each bundled meal (i.e., sum of main dish, side dish and beverage). All meals contain 0 g of trans fat. Nutrition information for individual food items is available upon request.
